# Influence of blood-brain barrier and intestinal barrier on microglial phenotypes: a perspective of clinical and prognostic implications

**DOI:** 10.3389/fimmu.2026.1663796

**Published:** 2026-05-19

**Authors:** Maiara N. Lima, Lucas F. Santos, Felipe Simões Lemos, Patricia Alves Reis, Tatiana Maron-Gutierrez, Hugo C. Castro-Faria-Neto

**Affiliations:** 1 Centro de Pesquisa, Inovação e Vigilância em Covid-19 e Emergências Sanitárias, Oswaldo Cruz Institute, Oswaldo Cruz Foundation, Rio de Janeiro, Brazil; 2 Biochemistry Department, State University of Rio de Janeiro, Rio de Janeiro, Brazil

**Keywords:** biological barrier, blood-brain barrier, intestinal barrier, microglia, microglial phenotype, neurological disorders

## Abstract

Microglia are highly specialized immune cells of the central nervous system (CNS). These cells exhibit remarkable plasticity, enabling them to adopt distinct phenotypes and functional profiles in response to changes in their microenvironment. Biological barriers, such as the blood-brain barrier (BBB) and the intestinal barrier, play a crucial role in maintaining the organism’s homeostasis, protecting organs and tissues against pathogens, toxins, and other external factors. Alterations in the permeability of the BBB and the intestinal barrier expose microglia to peripheral molecules, such as cytokines, LPS, and bacterial metabolites. These molecules reach the CNS via systemic circulation, activating microglia through Toll-Like Receptor (TLR) and Nod-like receptor family pyrin domain-containing (NLRP) receptors and lead to a transition in microglial phenotype. Elucidating bidirectional communication between these barriers and microglia holds significant potential for developing novel therapeutic targets, particularly in neurological disorders with limited treatment options. In this perspective, we discuss how alterations in the BBB and intestinal barrier affect microglial phenotypes, contributing to the development of neurological disorders.

## Introduction

Microglia are the resident immune cells of the central nervous system (CNS), which are essential for the refined development and functioning of the CNS throughout embryonic periods and life. Distinct from other glial cell types, microglia have a hematopoietic origin, distinguishing them from other glial cells originating from the neuroectoderm (1, 2). In contrast to other glial cells, and similar to peripheral immune cells, microglia exhibit a high sensitivity to inflammatory stimuli (3, 4). Moreover, microglia are among the earliest cellular responders to alterations in the CNS microenvironment, largely due to their extensive repertoire of pattern-recognition and cytokine receptors that enable rapid sensing of danger and inflammatory signals (5, 6). This high cellular responsiveness gives rise to distinct phenotypic variations, each assuming specific functional roles that are crucial for maintaining homeostasis and responding to pathological conditions.

Microglial plasticity depends on the stability of the CNS microenvironment, which is strictly regulated by biological barriers that are essential to its proper functioning. Biological barriers act not merely as physical walls, but as dynamic gatekeepers that control the signaling landscape in which microglia reside (7, 8). Biological barriers, such as the BBB, intestinal barrier, and blood-spinal cord barrier, are essential to the physiological functioning of the CNS. The dysfunctions in these biological barriers can modulate the microenvironment and alter microglial phenotype, potentially exacerbating neuroinflammatory and neurodegenerative processes (9). Understanding the impact of biological barriers on microglial phenotypes is crucial for developing targeted therapeutic strategies for these processes. This perspective aims to explore the interplay between biological barriers and microglial phenotypes, highlighting their clinical and prognostic implications.

## The dynamic role of microglia and its functional plasticity

Microglia exhibit high functional plasticity, as these cells change their phenotype and function in response to the microenvironment. The concept of microglial phenotypic plasticity was previously defined in binary terms, with these cells classified as “active” or “inactive”, with the “active” state associated with the progression of CNS damage. The term “microglial polarization” (M1/M2) has also been criticized as overly simplistic and reductionist (10). The development of new technologies enabled advances in neuroscience, revealing that microglia are not confined to just two static states; instead, they are highly plastic cells exhibiting a diverse range of functional profiles characterized by varying cellular and molecular responses (10).

Given the wide phenotypic spectrum of microglia states, it remains challenging to establish a clear nomenclature for each phase. David Gosselin and Christopher Glass, pioneers in this field, demonstrated the existence of a spectrum of microglial phenotypes with distinct transcriptional and functional states (11). The shift occurs dynamically in response to microenvironmental stimuli, allowing these cells to play either protective or neurotoxic roles. Within the phenotypic spectrum of microglia, the homeostatic, pro-inflammatory, anti-inflammatory, primed, and senescent states are terms currently well accepted. Besides that, new studies are being developed, and new nomenclatures have been proposed and are increasingly accepted, including disease-associated microglia (DAM), originally described in Alzheimer’s disease (AD) but now recognized in other pathological conditions (12, 13); the microglial neurodegenerative phenotype (MGnD) (14); and activated response microglia (ARM) (15). Together, these classifications highlight how microglia adapt to injury, inflammation, and metabolic stress, reinforcing the idea of a flexible rather than static phenotypic landscape.

Microglial phenotyping has evolved substantially with the development of advanced molecular, imaging, and computational methodologies, enabling a more systematic resolution of microglial heterogeneity. Among these approaches, single-cell RNA sequencing (scRNA-seq) has emerged as a key method for high-resolution transcriptomic profiling of microglia. This technology allows high-resolution transcriptomic profiling at the single-cell level, enabling the unbiased identification of distinct microglial states under homeostatic and pathological conditions (16, 17). Notably, scRNA-seq led to the identification of DAM microglia, characterized by transcriptional programs regulated by genetic axes such as Triggering Receptor Expressed on Myeloid cells 2 (TREM2) and APOE (12). More broadly, scRNA-seq captures dynamic transitions between functional states and reveals context-dependent reprogramming in response to inflammation, neurodegeneration, or therapeutic interventions (17). Complementary high-dimensional approaches, including bulk RNA sequencing, spatial transcriptomics, and proteomic profiling, further refine phenotypic classification by integrating spatial and molecular information (18–20). These strategies allow genome-wide sampling and facilitate the identification of signaling pathways, metabolic shifts, and regulatory networks underlying microglial functional diversity.

Historically, microglial phenotyping relied predominantly on morphological criteria. The transition from a “ramified” to an “amoeboid” morphology was interpreted as a hallmark of “activation” (21). However, advances in high-throughput imaging pipelines and automated morphometric analysis now enable quantitative assessment of branching complexity, soma size, process length, and spatial distribution at the single-cell level. Importantly, accumulating evidence demonstrates that morphology alone is an unreliable proxy for functional state (22). Microglia displaying similar shapes may exhibit markedly distinct transcriptional and proteomic signatures depending on their microenvironment.

When microglia shift from their homeostatic phenotype to other states, they cease to play their essential role in maintaining the CNS physiology. This transition alters the brain’s microenvironment, leading to changes linked to pathological conditions (3, 4, 23–25). In the proinflammatory state, microglia cells can perform macrophage functions, including phagocytosis, cytokine production, and antigen presentation. Proinflammatory microglia have the Nuclear factor kappa B (NF-κB), Mitogen-activated protein kinase (MAPK), and JAK/STAT signaling pathways activated, which are consistent with the inflammatory response performed by these cells (4, 23). Hence, they can produce and release immune mediators, including reactive oxygen species, interleukin (IL)-1β, IL-6, tumor necrosis factor-α (TNF-α), nitric oxide, and other inflammatory molecules (26; Y. S. 3, 27, 28). The pro-inflammatory response aims to protect and repair neural tissue; however, when this response is prolonged or chronic, it can cause neurotoxicity, contributing to the progression of different pathological conditions (3, 23, 27).

Cellular metabolism underlies all biological processes by coordinating energy generation and the synthesis of essential biomolecules. Within the CNS, substrates such as glucose, fatty acids, and amino acids critically shape neuroimmune function by activating nutrient-sensing mechanisms and driving metabolic reprogramming in microglia (29). It has been demonstrated that, once pattern recognition receptors (PRRs) activate, metabolic states, such as glycolytic versus oxidative profiles, become central to microglial function, with relevant references supporting these distinctions (29, 30).

Microglial functional states are closely linked to immunometabolic remodeling, where pro-inflammatory states involve increased glycolysis, and homeostatic states depend on oxidative metabolism (29, 30). Metabolic changes, including increased Glut-1 receptors and glycolysis, highlight how pathways like glycolysis and OXPHOS influence microglial phenotypes, inspiring interest in their therapeutic targeting (29). The anti-inflammatory and reparative microglia rely on oxidative metabolism fueled by fatty acids and glucose, supporting tissue repair and inflammation resolution (29). Microglia glutamine metabolism is linked to the regulation of processes such as NLRP3 inflammasome, reflecting a metabolic function beyond mere energy generation (31). Recognizing how metabolic pathways govern microglial functions suggests that targeting specific metabolic programs could modulate microglial responses, offering promising avenues for therapeutic intervention in neurodegenerative diseases and CNS injuries (30).

Despite significant progress having been made in redefining microglial phenotypes beyond the classical M1/M2 model toward a dynamic and context-dependent spectrum of functional states, microglial phenotyping remains an active and evolving field. Contemporary microglial phenotyping increasingly integrates transcriptomic, proteomic, metabolic, and morphometric datasets, moving beyond simplistic activation paradigms toward multidimensional and context-dependent classification frameworks.

A major challenge lies in the substantial overlap among transcriptional markers across microglial subtypes, which hampers their functional discrimination and state classification. This underscores the need for more specific and functional markers, particularly those validated in human tissues. Current trends emphasize the characterization of condition-specific subtypes (e.g., disease-associated microglia) and the integration of microglial metabolic profiling. In addition, growing attention is being directed toward understanding how biological barriers, including the blood-brain and intestinal barriers, modulate microglial plasticity.

## Biological barriers as a potential driver of microglial phenotypes

The CNS is a highly protected and tightly regulated environment that maintains cerebral homeostasis through specialized structural and functional barriers. Among these, the BBB is a critical determinant of brain integrity (7, 32). BBB homeostasis is multifactorial and largely dependent on its highly specialized architecture. The barrier is formed by a tightly adherent endothelial layer sealed by tight junction proteins and supported by perivascular astrocytic end-feet, which contribute to barrier stability and regulation. (7). The BBB’s finely tuned structure regulates the entry of cells and molecules from the bloodstream into the CNS, including cytokines and reactive oxygen species (ROS). Beyond structural support, astrocytes actively participate in endothelial signaling and vascular homeostasis, thereby limiting microglial exposure to circulating inflammatory mediators. Under physiological conditions, this astrocyte-endothelial cooperation preserves CNS immune isolation and sustains homeostatic microglial gene expression profiles (33).

In response to microenvironmental changes, the BBB-associated cell types (endothelial cells, astrocytes, and microglia) undergo phenotypic and functional transitions (32). In pathological contexts, such as infectious diseases, ischemic or hemorrhagic stroke, psychiatric disorders, and intestinal dysbiosis, BBB dysfunction allows for an inflammatory infiltrate into CNS, exposing resident cells to different cytokine gradients and immune complexes (antibodies and complement system factors) (34). At the molecular level, endothelial cells of the BBB respond to inflammatory mediators, such as IL-1β, TNF-α, C-C motif chemokine ligand 2 (CCL2), and ROS, through the activation of intracellular signaling pathways, including NF-κB, MAPKs, and oxidative stress-responsive cascades (34–37). Activation of these signaling pathways promotes endothelial reprogramming, characterized by the disruption of tight junctions, increased expression of adhesion molecules, and the secretion of cytokines and chemokines into the perivascular niche. These endothelial-derived mediators are sensed by astrocytic end-feet and other components of the neurovascular unit, initiating a coordinated glial response. Reactive astrocytes may subsequently buffer or amplify inflammatory signaling by releasing additional cytokines and chemokines, thereby contributing to the propagation of neuroinflammatory responses and modulating microglial phenotype in a context-dependent manner (35, 38, 39). Thus, astrocyte responses to barrier dysfunction act synergistically to determine the magnitude and direction of microglial phenotypic transitions.

The BBB-associated astrocytes play a pivotal role in modulating microglial activity by releasing a wide repertoire of signaling molecules, such as ATP, glutamate, cytokines (e.g., IL-6, TGF-β), and chemokines (e.g., CXCL10, CCL2) (35, 38). In addition, astrocytes secrete extracellular vesicles containing mRNAs and other regulatory molecules that may influence microglial gene expression and functional states (40–43). These astrocyte-derived signals contribute to the dynamic modulation of microglial phenotypes, which may shift toward proinflammatory or neuroprotective profiles depending on the nature, intensity, and duration of BBB dysfunction.

Under conditions of BBB dysfunction, microglia, initially in a homeostatic state, switch to a pro-inflammatory phenotype to resolve the damage. Persistent BBB dysfunction can drive microglia toward persistent dystrophic phenotypes, characterized by impaired phagocytic activity and a reduced capacity for homeostatic surveillance (9, 43, 44). Conversely, transient or regulated BBB-derived signals can promote reparative microglial phenotypes, such as DAM and other context-specific adaptive states, which contribute to debris clearance and tissue remodeling (9, 45). However, in some cases, prolonged exposure to proinflammatory mediators can further favor the development of primed microglial states, characterized by heightened sensitivity to secondary stimuli and exacerbated responses, increasing brain vulnerability (46–48).

Communication between microglia and the BBB is bidirectional and tightly regulated. Although BBB dysfunction represents the initial trigger for microglial response in many pathological contexts, the inverse sequence may also occur, whereby microglial activation precedes and contributes to BBB impairment (9, 49, 50).

The accumulation of misfolded or aggregation-prone proteins, such as β-amyloid, α-synuclein, and hyperphosphorylated tau, within the brain parenchyma can be directly sensed by microglia (51–55). Similarly, local metabolic or homeostatic disturbances, including chronic hyperglycemia, mild or intermittent hypoxia, and excessive levels of excitatory neurotransmitters (e.g., glutamate), can lead to primary microglial response (56–60). These microenvironmental brain changes are recognized by microglia via PRRs, including TLRs, TREM2, and NLRP3. Activation of these pathways initiates a proinflammatory cascade that promotes the production and release of inflammatory mediators, which can, in turn, compromise BBB integrity (23, 55, 61, 62). In this context, intrinsic alterations in the brain parenchyma precede BBB dysfunction, and the resulting microglia-driven inflammatory response may secondarily disrupt BBB structure and function. Therefore, BBB integrity and the dynamic signaling interactions between the barrier and microglia are critical determinants of CNS homeostasis and function.

Besides the BBB, peripheral barriers, such as the intestinal barrier, seem to be directly associated with brain function, playing a role in either maintaining homeostasis or contributing to illness of this organ (63, 64). The intestinal barrier is functionally maintained by mucus secreted by intestinal glands and goblet cells, as well as by tightly attached enterocytes with their occluding junctions. The rupture of this barrier exposes the connective tissue, known as the lamina propria, which is highly vascularized and allows luminal molecules to enter the bloodstream.

Growing evidence suggests that gut dysbiosis is directly linked to microglial response and their transition to pro-inflammatory phenotypes, mediated by alterations in intestinal barrier integrity (8, 63, 65–67). The gut microbiota is involved in the bidirectional regulation of the intestine and CNS through neural, endocrine, metabolic, and immunological pathways (65). The study by Lynch et al. demonstrated that disrupting the gut microbiota through antibiotic administration in neonatal mice during critical windows of early development induces morphological changes in microglia (68). In conditions such as Alzheimer’s, autism spectrum disorder and depression, individuals frequently exhibit dysbiosis that is related to leaky gut (69–72). Alterations in the intestinal microbiota impair the intestinal mucosal barrier, increasing intestinal permeability and facilitating the release and translocation of endotoxins, such as lipopolysaccharide (LPS), into the peripheral blood circulation. Other microbial metabolites could promote microglial responses; for example, increased levels of gut isoamylamine cross the BBB and induce microglial apoptosis in an aging experimental model (73). Leclercq and colleagues demonstrated that anxiety, depression, and craving are directly related to intestinal permeability during alcohol use disorder (74). Increased intestinal permeability and the subsequent inflammatory response promote BBB dysfunction and neuroinflammation, thereby impairing neurological function.

Microglia are a crucial cell type in the bidirectional communication of the gut-brain axis. During chronic stress, both microglia and the intestinal barrier are affected, and direct therapeutic intervention targeting the microbiota can restore the homeostatic microglial profile (75). Recently, it has also been demonstrated that dietary enrichment with bovine milk increases the microglial population in the cerebral cortex and cerebellum, together with intestinal alterations (76). However, at our current level of knowledge, there is no canonical pathway that governs this communication. BBB permeability seems to be one of the major ways that dysbiosis, joined by intestinal permeability, leads to microglia dysfunction (77).

The spinal cord is part of the CNS and originates from the neural tube. It receives sensory nerves and projects motor axons to the periphery. In composition, the spinal cord is similar to the brain, containing glial cells and numerous neurons. This structure also protects the CNS from circulating blood molecules. Recently, growing evidence indicated that there is also a bidirectional communication pathway between the spinal cord and the gut. The removal of intestinal microbiota prevents allodynia in a mouse model of nerve injury. Interestingly, microbiota replacement restores allodynia. TNF-α production is reduced in antibiotic-treated mice and restored after microbiota reestablishment, contributing to chronic pain (78). In rat models of spinal cord injury, physical exercise combined with fecal transplantation reduces intestinal and spinal cord inflammation and reduces microglial inflammatory activity in damaged tissue (79).

**Figure 1:**
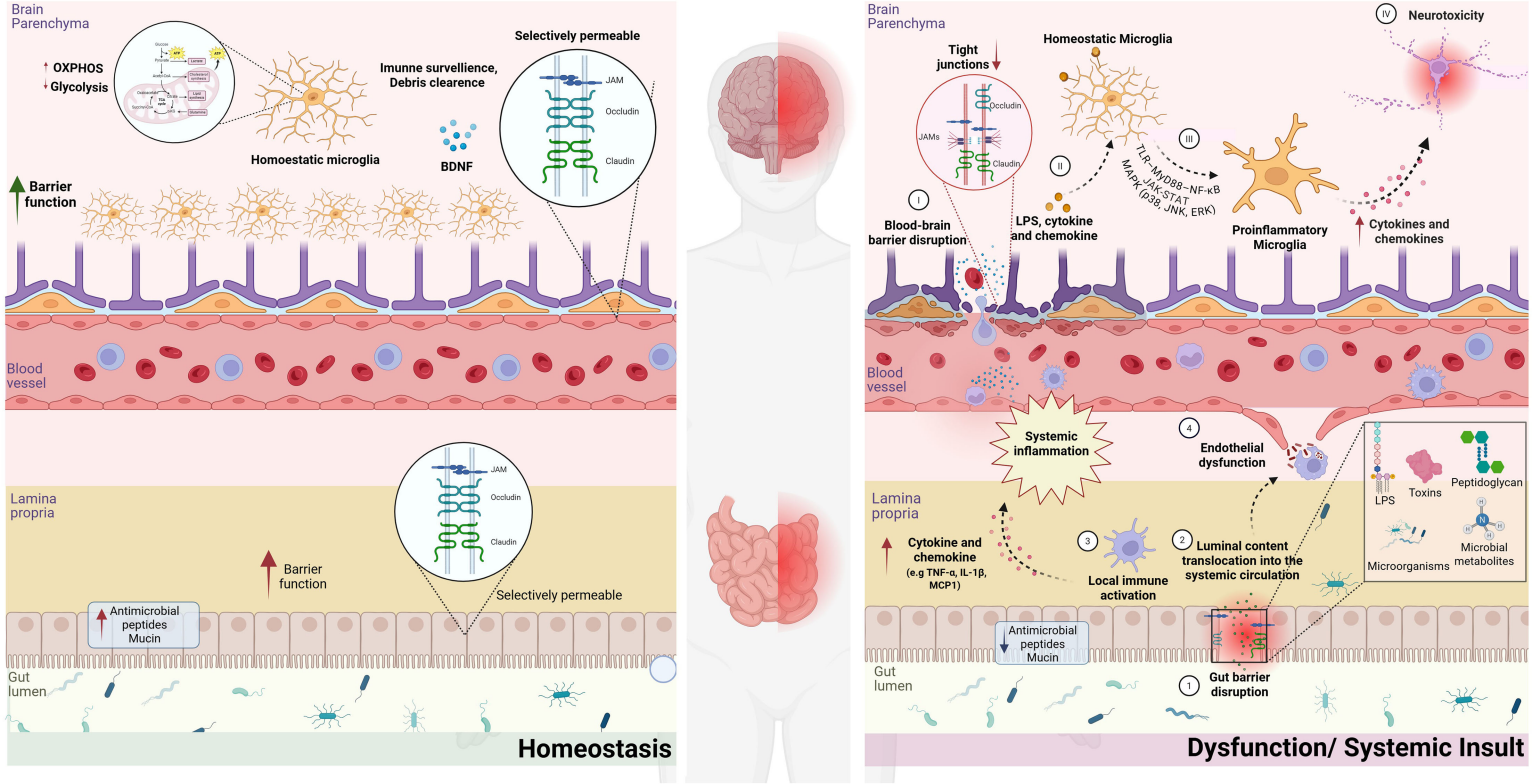
The blood-brain and intestinal barriers dysfunction as a factor in microglial phenotypic transition. The left panel depicts the homeostatic condition in which the intestinal barrier and the blood-brain barrier (BBB) preserve structural and functional integrity through properly organized tight junction proteins (claudins, occludin, and JAMs), ensuring immune compartmentalization between the peripheral circulation and the brain parenchyma. Under these conditions, microglia maintain a homeostatic phenotype characterized by immune surveillance, debris clearance, neurotrophic support (e.g., BDNF release), and balanced metabolic integration of glycolysis and oxidative phosphorylation (OXPHOS), thereby sustaining central nervous system (CNS) stability. The right panel highlights microglial phenotypic transition as the central event triggered by biological barrier dysfunction. In the gut, intestinal barrier disruption (1) permits luminal content translocation into the systemic circulation (2), triggering local immune activation and systemic inflammation (3), which contributes to endothelial dysfunction (4). In the brain, BBB disruption (I) enables peripheral inflammatory mediators and immune cells to access the brain parenchyma. These signals are sensed by microglia through pattern recognition receptors (II), activating intracellular pathways including TLR-MyD88-NF-κB, JAK/STAT, and MAPKs (III). This signaling cascade drives the transition from a homeostatic to a pro-inflammatory microglial phenotype. The phenotypic shift is associated with enhanced production of inflammatory mediators, metabolic reprogramming, reduced neuroprotective functions, and increased neurotoxicity (IV). Collectively, the figure highlights biological barrier dysfunction as a mechanism linking microglial phenotypic transition. Created in BioRender. https://BioRender.com/ocfwb9e.

Collectively, current evidence supports a dynamic crosstalk between biological barriers and microglial phenotypic states (**Figure 1**), highlighting their integrated role in shaping the CNS microenvironment. In this context, understanding how barrier-mediated signals regulate microglial functional states across homeostatic and disease conditions is essential to delineate microglial phenotypic trajectories and their association with disease progression.

## From homeostasis to disease: microglial phenotypic transitions and prognostic implications

Under homeostatic conditions, biological barriers maintain a tightly regulated CNS microenvironment that supports microglial surveillance functions, including synaptic monitoring, debris clearance, and trophic support. In contrast, disruption of this regulation alters the balance of incoming signals, shifting microglia toward phenotypes associated with inflammatory signaling, metabolic reprogramming, and impaired homeostatic functions (**Figure 1**^80^, ^81^).

Across pathological contexts, a common feature is the loss of barrier-controlled protection, leading to increased exposure of the CNS to peripheral inflammatory cues and local neurovascular signals, which reshape microglial functional states. In sepsis, systemic inflammation enables the influx of circulating mediators and microbial-derived products in the CNS, driving microglial reprogramming and contributing to long-term cognitive impairment (82, 83). A comparable systemic-to-central signaling cascade is observed in cerebral malaria, where vascular dysfunction, endothelial activation, and peripheral inflammatory amplification converge to promote sustained neuroimmune mechanisms (84, 85).

Chronic alterations in barrier-regulated signaling are associated with the emergence of microglial DAM phenotype (12, 34, 86). This phenotype is characterized by TREM2–APOE-driven transcriptional reprogramming, accompanied by the loss of homeostatic markers (e.g., P2RY12, TMEM119) and the upregulation of genes involved in lipid metabolism and phagocytosis (e.g., LPL, CST7, SPP1) (87). Within this context, sustained exposure to peripheral and local cues reinforces maladaptive microglial programs and impairs clearance of pathological protein aggregates, contributing to disease progression (88).

Acute neurological insults, including ischemic stroke and traumatic brain injury, further illustrate how rapid disruption of vascular integrity reshapes barrier-mediated signaling, exposing the CNS to circulating factors and amplifying neurovascular unit-derived cues that drive the microglial phenotypic shift. The magnitude and persistence of these alterations correlate with clinical severity and neurological outcomes (34). In parallel, systemic conditions such as depression and metabolic disorders highlight how disturbances in gut barrier homeostasis can indirectly influence CNS signaling environments, further modulating microglial phenotypes (65, 69).

Collectively, these observations support the notion that barrier-regulated signaling dynamics are key determinants of microglial phenotypic trajectories across diseases. Importantly, the duration and intensity of these perturbations may carry prognostic value, as sustained dysregulation is consistently associated with maladaptive microglial states and worse clinical outcomes (89, 90). In this context, biological barriers emerge not only as regulators of disease progression but also as strategic targets for modulating microglial responses, providing a conceptual basis for the development of targeted therapeutic interventions (91).

## Protecting biological barriers: potential therapeutic strategies to reduce neuroinflammation

Modulation of the microglial phenotype may be an effective strategy for treating various neuroinflammatory pathologies. Mitigating the biological barriers dysfunction can control microglial pro-inflammatory activity related to neurologic impairments (32, 92). Therapeutic strategies that modulate the permeability of biological barriers, strengthen endothelial junctions, and consequently modulate neuroinflammation may be of great relevance in this context.

Substances that modulate the expression of proteins such as occludin, claudins and Zonula Occludens-1 (ZO-1) may reinforce the BBB and prevent changes in the homeostatic microglial phenotype, protecting against pathological conditions. Netrin-1, a protein involved in cell migration and axon guidance during development, downregulates claudin-5, occludin, and ZO-1 expression induced by traumatic brain injury (93). Statins (simvastatin and atorvastatin) protect the BBB after experimental intracerebral hemorrhage. Statin-treated animals showed reduced BBB permeability, and occludin expression was increased after treatment (94). In addition, statins have been shown to reduce leukocyte adhesion to brain vessels, thereby reducing edema development in experimental models of cerebral malaria and sepsis (95, 96) and improve capillary density and function in a rat model of hypertension (97). Therefore, therapeutic strategies that modulate BBB tight junctions may aid in the proper functioning of the BBB in pathogenic conditions. These strategies may involve direct gene editing to restore junction protein expression, using pharmacological approaches, CRISPR/Cas9, viral vectors, small interfering RNA (siRNA), micro ribonucleic acid (miRNA), or bioactive natural compounds.

Innovative approaches, such as cell therapy and nanoparticles, are emerging as promising alternatives to control biological barrier dysfunction and modulate proinflammatory microglial phenotypes. Intravenous mesenchymal stromal cells (MSCs) therapy reduced the BBB dysfunction and astrogliosis in mice in an experimental model of sepsis (98). This may correlate with the suppression of peripheral inflammation, as a reduction in plasma proinflammatory mediators was also observed in septic animals. MSCs also reverse the BBB disruption and peripheric damages in an experimental malaria model (99). Peripheral inflammation can modulate microglia phenotype through various immunoneural mechanisms. The control of peripheral inflammation by MSCs treatment may have an influence on the microglial phenotype. In an experimental stroke model in mice, the intravenous administration of nanoencapsulated IL-10 mRNA in a microglia-targeting lipid system engineered to recognize the CD206 receptor, which enhances the homing of the nanoparticles to ischemic regions of the brain, restores the impaired BBB and induces the microglia production of IL-10 (100).

Several groups are engaged in a race to discover approaches that can reduce intestinal permeability and, consequently, modulate microglia. Short-chain fatty acids (SCFAs), probiotics, fecal transplantation, and vaccines are considered future clinical approaches. Clinical and preclinical studies have demonstrated that fecal microbiota transplantation can significantly alleviate neurological disorders and act as a protective treatment against neuroinflammation (101). Transplantation of gut microbiota from healthy donors reduced serum concentrations of 5-hydroxytryptamine (5-HT) and γ-aminobutyric acid (GABA) in autistic children, while dopamine levels increased 4 weeks after transplantation (102). These neurotransmitters can have pro- or anti-inflammatory effects on microglia, depending on the type of receptor activated. GABA exerts immunoregulatory action, reducing the pro-inflammatory activity of microglia (M. 103, 104). Dopamine has anti-inflammatory effects on microglia, especially through the activation of D1 and D2 receptors, which suppress pathways such as NF-κB and reduce the release of pro-inflammatory cytokines (105). Recent evidence demonstrates that *Bifidobacterium longum* alleviates depressive-like behavior in mice by increasing gut-derived homovanillic acid (HVA) production, a dopamine metabolite. Given that microglia express dopamine receptors, this may influence their response. The treatment also enhanced hippocampal mBDNF expression, a neurotrophin known to modulate microglial behavior via TrkB signaling, promoting a neuroprotective phenotype. (106). Together, these findings support the notion that alterations in intestinal barrier permeability and microbiota can influence microglial phenotypes.

Butyric acid (BA) is one of the most abundant SCFAs in the gut, produced in the intestinal microbiota by symbiotic bacteria as well as ingested through various foods (milk, butter, and cheese). BA is absorbed by enterocytes, used as an energy source, and can also be translocated to connective tissue before finally entering the bloodstream (67). Dietary supplementation with BA improved alcohol-induced neurological damage by suppressing microglia-mediated neuroinflammation via the GPR109A/PPAR-γ/TLR4-NF-κB signaling pathway and modulating the gut-brain microbiota axis (107). However, the BA concentrations capable of reaching the brain are small. According to studies in primates, only 0.006% of the administered conjugated butyrate reached the CNS, with only 20% remaining after 5 minutes (108). Different cell types, such as hepatocytes, enterocytes, neurons, and astrocytes, express distinct SCFAs transporters. One of these transporters, Monocarboxylate Transporter 1 (MCT1) (H^+^-coupled monocarboxylate transporter), plays a crucial role in BA passage across the BBB. In the LS174T intestinal epithelial cell line, BA treatment reduced proliferation and altered cell morphology. Data also indicate that the treatment leads to a dose-dependent increase in mucin-2, a protein that forms the mucus barrier and protects the intestinal epithelium from direct contact with pathogens (109). Mice subjected to a high-fat diet protocol conventionally develop increased intestinal permeability and fatty liver disease. High-fat diet treatment supplemented with BA reduces liver damage, increases intestinal ZO-1 mRNA expression, and expands its distribution along intestinal villi. In addition, reducing the expression of genes such as Monocyte Chemoattractant Protein-1 (MCP-1) and TNFα (110). In the mouse ileum, mRNA expression of occludin, claudin-4, and Muc20 are significantly higher in the presence of IL-1β + BA compared to IL-1β alone. Modulation of the gut microbiota through dietary intervention has also been shown to be effective in diseases such as Alzheimer’s and Parkinson’s, which cause BBB dysfunction (111). Furthermore, non-pharmacological interventions, such as regular physical exercise and dietary strategies rich in antioxidants and polyphenols, have demonstrated beneficial effects on microglial plasticity, contributing to the restoration of neuroimmune balance (111–114).

Since communication between biological barriers and microglia is bidirectional, reprogramming the phenotype and metabolism of microglia during disease may also be an effective strategy. Neuroprotective compounds, including flavonoids, curcumin, and omega-3 fatty acids, have shown potential in regulating microglial inflammatory role through the modulation of oxidative stress and anti-inflammatory signaling mediated by NRF2 and Peroxisome proliferator-activated receptor gamma (PPAR-γ) (113, 115–117). Molecules that modulate pathways related to the activation of TLRs receptors may be a therapeutic approach in the modulation of microglial proinflammatory metabolism. Inhibition of the NF-κB pathway by isoorientin, a molecule that can be extracted from several plant species, resulted in reduced expression of Inducible Nitric Oxide Synthase (iNOS) and cyclooxygenase-2 (COX-2) and inhibited the secretion of proinflammatory cytokines and apoptosis of BV2 cells induced by the stimulation of Aβ_25–35_ (118).

Another potential therapeutic approach for several CNS pathologies is to suppress microglial priming during the disease period. A multifaceted approach to neutralize microglial priming may be beneficial. However, this field of research remains in its early stages, and evidence remains limited. Knowledge of molecular markers that identify the primed state of microglia has contributed to the field’s development. Increased expression of certain genes and proteins related to the inflammatory response, such as CD11b, CD68, Ionized calcium-binding adapter molecule 1 (Iba-1), and TREM2, has already been observed. In preclinical models, beneficial results have been observed using minocycline, glucocorticoids, and antioxidants (47, 48). A summary of the therapeutic strategies discussed in this section is presented in **Table 1**.

**Table 1:** Therapeutic strategies targeting biological barriers and microglial modulation.

Pharmacological approaches					Non-pharmacological approaches				
Intervention	Therapeutic class	Molecular/Cellular mechanism	Primary effect on BBB, intestinal barrier, or microglia	References	Intervention	Therapeutic class	Molecular/Cellular mechanism	Primary effect on BBB, intestinal barrier, or microglia	References
Statins (simvastatin, atorvastatin)	Small-molecule drugs	Upregulation of tight junction proteins (e.g., occludin); reduced leukocyte adhesion; anti-inflammatory signaling	Reduced BBB permeability; attenuation of neuroinflammation	(94–97)	Fecal microbiota transplantation (FMT)	Microbiota modulation	Restoration of gut microbial balance	Reduced intestinal permeability; indirect microglial modulation	(101, 102)
Mesenchymal stromal cells (MSCs)	Cell therapy	Suppression of peripheral inflammation; immunomodulation	Reduced BBB dysfunction	(98, 99)	Probiotics (e.g., Bifidobacterium longum)	Microbial therapy	Increased production of neuroactive metabolites (e.g., homovanillic acid); increased mBDNF	Promotion of neuroprotective microglial phenotype	(106)
Netrin-1	Recombinant protein	Regulation of claudin-5, occludin, and ZO-1 expression	Preservation of BBB structural integrity after injury	(93)	SCFA-producing diets	Dietary intervention	Increased endogenous SCFA production; enhanced mucin and tight junction expression	Strengthened intestinal barrier; indirect microglial modulation	(110)
Nanoencapsulated IL-10 mRNA	Nanoparticle-based immunotherapy	Targeted induction of IL-10 expression in microglia (CD206-mediated targeting)	BBB restoration; induction of anti-inflammatory microglial phenotype	(100)	Physical exercise	Lifestyle intervention	Anti-inflammatory signaling; improved vascular and metabolic function	Enhanced microglial plasticity; improved BBB function	(112, 114)
Butyric acid (BA)	Short-chain fatty acid	Modulation of GPR109A/PPAR-γ/TLR4–NF-κB pathway	Reduced microglia-mediated neuroinflammation; improved intestinal barrier integrity	(107–110)	Antioxidant- and polyphenol-rich diets	Nutritional strategy	Activation of NRF2; reduction of oxidative stress	Restoration of neuroimmune balance	(111)
Isoorientin	Flavonoid	Inhibition of NF-κB signaling; decreased iNOS and COX-2 expression	Reduced pro-inflammatory microglial response	(118)					
Curcumin	Polyphenol	Activation of NRF2 and PPAR-γ pathways; antioxidant activity	Promotion of neuroprotective microglial phenotype	(115, 116)					
Omega-3 fatty acids	Lipid mediators	Regulation of oxidative stress and inflammatory signaling	Modulation of microglial inflammatory response	(113, 117)					
Minocycline	Tetracycline antibiotic	Inhibition of pro-inflammatory microglia and priming	Reduction of neuroinflammatory responses	(47, 48)					
Glucocorticoids	Steroidal anti-inflammatory drugs	Suppression of pro-inflammatory gene transcription	Attenuation of microglial priming	(47, 48)					
Antioxidants	Redox modulators	Reduction of oxidative stress pathways	Modulation of microglial pro-inflammatory state	(47, 48)					

BBB, blood-brain barrier; SCFA, short-chain fatty acid; MSC, mesenchymal stromal cell; siRNA, small interfering RNA; miRNA, microRNA; iNOS, inducible nitric oxide synthase; COX-2, cyclooxygenase-2; NRF2, nuclear factor erythroid 2-related factor 2; PPAR-γ, peroxisome proliferator-activated receptor gamma.

## Future directions and perspectives

Combining pharmacological approaches (e.g., statins) with non-pharmacological strategies, including MSC therapy and lifestyle-based interventions (e.g., regular physical activity), has shown promising effects. However, despite the growing number of strategies targeting barrier protection and microglial modulation, important challenges remain. Most available data derive from animal models, with limited insight into long-term outcomes and translational relevance in humans. Furthermore, many current therapies targeting tight junctions or inflammatory pathways lack cellular specificity, raising the risk of systemic side effects.

Bridging the gap between mechanistic insight and clinical translation is both a major challenge and a critical opportunity. The roles of barrier-derived extracellular vesicles and microglial metabolic signaling in shaping phenotypic responses remain poorly characterized and represent a particularly promising area for investigation. Future research should prioritize integrative models that combine advanced imaging, multi-omics platforms, and systems biology approaches to decode the bidirectional communication between microglia and biological barriers. This could lead to the identification of novel regulatory pathways, facilitate the discovery of early biomarkers of barrier dysfunction, and guide the development of more precise, barrier-targeted interventions.

The development of microglia- or endothelium-specific delivery systems, such as ligand-targeted nanoparticles, offers a powerful strategy for selectively delivering therapeutic molecules, including drugs, RNAs, and proteins. By conjugating targeting ligands (e.g., peptides or antibodies) to their surface, these nanoparticles enable cell-specific uptake, thereby minimizing off-target effects and reducing systemic toxicity. Gao et al. employed nanoparticles functionalized with ligands targeting the mannose receptor (CD206), a surface molecule highly expressed by microglia, particularly those with an anti-inflammatory phenotype. Similarly, mannose-coated nanoparticles loaded with a miR-17 antagomir have demonstrated therapeutic benefits in a preclinical model of Alzheimer’s disease (119). Cell-targeted nanoparticles represent a promising therapeutic avenue for restoring barrier integrity and maintaining CNS homeostasis. This strategy can be further refined through gene-editing tools, which may enhance treatment specificity and efficacy. Tools such as CRISPR/Cas9 and RNA interference (RNAi) allow targeted silencing or activation of genes involved in microglial phenotype (e.g., TREM2, NF-κB, NLRP3) and barrier integrity (e.g., claudin-5, occludin, ZO-1).

Altogether, these strategies represent a promising frontier for reprogramming microglial-biological barrier communication in a context- and disease-specific manner, opening avenues for precision therapies in CNS disorders.

## Conclusion

The interplay between biological barriers and microglial phenotypes represents a critical frontier in neuroinflammation research. Unravelling how context-specific microglial states interact dynamically with distinct barrier systems is essential to elucidate the mechanisms that drive disease progression. This line of investigation also holds promise for identifying convergent regulatory pathways and novel, highly specific therapeutic targets.

Among the most promising avenues for future research is the development of cell-type-specific delivery systems, particularly those targeting microglia and endothelial cells. Strategies such as ligand-directed nanoparticles and gene-editing methodologies offer considerable potential to overcome current limitations in cellular specificity, enabling precise modulation of neuroimmune and barrier-related responses in a disease- and context-dependent manner.

Therefore, integrating the study of barrier dysfunction into the broader framework of microglial biology could not only reshape our understanding of neuroinflammation but also pave the way for disease-modifying therapies in a wide range of CNS disorders.
